# T1 Healthcare Utilization After Thermal Injury: An Analysis Using a Commercial Claims Database

**DOI:** 10.1093/jbcr/irac012.000

**Published:** 2022-03-23

**Authors:** Aislinn E Lewis, Joshua J Horns, Irma D Fleming, Giavonni M Lewis, Callie M Thompson

**Affiliations:** Univerisity of Utah, Salt Lake City, Utah; University of Utah, Salt Lake City, Utah; University of Utah Burn Center, Salt Lake City, Utah; University of Utah Burn Center, Salt Lake City, Utah; University of Utah Health, Salt Lake City, Utah

## Abstract

**Introduction:**

Burn Injuries directly affect more than 500,000 people per year in the United States but no previous studies have looked at the impact on the healthcare system. Several prior studies have shown decrease in unplanned healthcare utilization after acute care discharge following burn injury and increased healthcare costs in patients who have undergone grafting for burn injury. The goal of this study is to describe post-acute care hospitalization healthcare use in burn patients.

**Methods:**

A retrospective commercial claims database review was performed of the Truven MarketScan (MS) database. In a 10 year MS sample, 23,262 patients with burn injuries were identified and were matched to a control population in a 1:1 ratio based on age, sex, and total time in the MS database. For the burn patient population in the study, pre-burn and post-burn utilization of therapy, emergency department, nutritional support, psychiatry/psychology, home health, skilled nursing facility, inpatient, and outpatient visits were recorded. For controls, we defined their pre-burn and post-burn periods using the burn event date of the matched case adjusted by the pair’s relative difference in initial enrollment into the MS database, and then recorded the same utilization metrics. A series of negative binomial regressions were completed to evaluate the data.

**Results:**

At the conclusion of the study, for every outcome, except skilled nursing facility, healthcare utilization was greater in the pre-injury burn group relative to controls. Healthcare utilization for the burn cohort post-injury was greater for every outcome compared to controls. Relative to controls, healthcare utilization remains higher for at least 25 months post-injury in the burn patients and does not return to pre-injury levels during this time frame.

**Conclusions:**

In a commercial claims database study, the healthcare utilization of the burn patients is higher both before and after burn injury than matched controls. Utilization does not return to preburn levels in the 25-month follow-up period. Additionally, burn patients have higher healthcare utilization prior to injury compared to matched controls, which may indicate an important difference in baseline health of these patients, and an opportunity for injury prevention.

